# Roles of Rack1 Proteins in Fungal Pathogenesis

**DOI:** 10.1155/2016/4130376

**Published:** 2016-08-30

**Authors:** Xue Zhang, Rashmi Jain, Guotian Li

**Affiliations:** ^1^Department of Botany and Plant Pathology, Purdue University, West Lafayette, IN 47907, USA; ^2^Department of Plant Pathology and the Genome Center, University of California, Davis, CA 95616, USA; ^3^Feedstocks Division, Joint BioEnergy Institute, Lawrence Berkeley National Laboratory, Berkeley, CA 94720, USA; ^4^Biological Systems and Engineering Division, Lawrence Berkeley National Laboratory, 1 Cyclotron Road, Berkeley, CA 94720, USA

## Abstract

Pathogenic fungi cause diseases on various organisms. Despite their differences in life cycles, fungal pathogens use well-conserved proteins and pathways to regulate developmental and infection processes. In this review, we focus on Rack1, a multifaceted scaffolding protein involved in various biological processes. Rack1 is well conserved in eukaryotes and plays important roles in fungi, though limited studies have been conducted. To accelerate the study of Rack1 proteins in fungi, we review the functions of Rack1 proteins in model and pathogenic fungi and summarize recent progress on how Rack1 proteins are involved in fungal pathogenesis.

## 1. Introduction

Diseases caused by pathogenic fungi pose a serious threat to human, animal, plant, and ecosystem health [1, 2]. Particularly, plant diseases caused by fungi, for example, rice blast and wheat rust, have long been known as widespread threats to global food security [1, 3]. In pathogenic fungi, key proteins and signal transduction pathways play conserved roles in the regulation of different developmental and infection processes and are therefore valuable targets for controlling these devastating diseases [4, 5].

Heterotrimeric G proteins are involved in environmental sensing of nutrients, pheromones, stresses, and other stimuli in eukaryotes [6]. The heterotrimeric G protein complex consists of G*α*, G*β*, and G*γ* subunits. In the signal transduction pathway, heterotrimeric G proteins are activated by G protein-coupled receptors (GPCRs) that perceive various signals. In the absence of a stimulus, the GDP-bound G*α* monomer and the G*βγ* dimer form an inactive complex with the GPCR. Binding of the ligand to the GPCR triggers exchange of GDP with GTP in association with G*α* and subsequent dissociation of G*α* from G*βγ*, and the released active form of the G*βγ* dimer interacts with other effector proteins to activate downstream signal transduction pathways. One of them is the cyclic adenosine 3′,5′-monophosphate/protein kinase A (cAMP/PKA) pathway. The GPCR activates the adenylyl cyclase, which catalyzes the formation of cAMP that in turn activates the PKA-mediated signaling cascades. cAMP is degraded by the cyclic nucleotide phosphodiesterase, which, together with the adenylyl cyclase, balances the level of cAMP in the cell [7]. Another pathway activated by the GPCR is the mitogen-activated protein kinase (MAPK) pathway, consisting of three sequentially activating kinases, with the upstream MAPK kinase kinases (MAPKKKs) perceiving signals from the receptors and activating MAPK kinases (MAPKKs) that in turn activate the MAPKs through phosphorylation, for example, Fus3/Kss1 and Slt2 in* Saccharomyces cerevisiae* [8]. The phosphorylated MAPKs activate related transcription factors, which in turn regulate genes in different biological processes.

Receptor for activated C kinase 1 (RACK1) was originally identified as the binding protein for activated protein kinase C [9, 10]. Rack1 belongs to the WD-repeat-containing proteins and contains seven WD40 repeats. These seven WD40 repeats assemble into a typical seven-bladed *β*-propeller structure that provides an interactive platform for the binding of other proteins [11]. Rack1 proteins are constitutively expressed in eukaryotes. Rack1 has been intensively studied in mammalian cells [12, 13]. Rack1 functions in angiogenesis, tumor growth, cell migration, apoptosis, autophagy, neuronal response, proper function of the circadian clock, defense responses against virus infection, chromatin remodeling, transcriptional and translational regulations, and cAMP/PKA and MAPK pathways [11–15]. Rack1 regulates these biological processes through the control of protein complex assembly [13]. In plants, Rack1 proteins are functional in seed germination, leaf production, flowering, hormonal signaling, and biotic/abiotic stress responses [16–18]. The emerging role of Rack1 as a scaffold protein in the MAPK pathway in* Arabidopsis thaliana* has been recently published [19–21]. In protozoan parasites, Rack1 proteins are critical for effective mammalian parasitization by* Leishmania major* and* Trypanosoma brucei*, which cause leishmaniasis and African trypanosomiasis, respectively [22–24]. The* L. major* LACK (*Leishmania* homologue of mammalian RACK) antigen is also a target of the immune response in mice and therefore a vaccine candidate for human leishmaniasis [22]. In* Plasmodium falciparum*, the most lethal malarial parasite, the Rack1 protein inhibits Ca^2+^ signaling of the mammalian cells and subverts the host intracellular environment, an important mechanism for parasite survival [25]. In summary, Rack1 proteins play important roles across a wide variety of organisms. In this review, we summarize recent progress on Rack1 proteins in model and pathogenic fungi.

## 2. Rack1 Homologs Are Well Conserved

**Figure 1:**
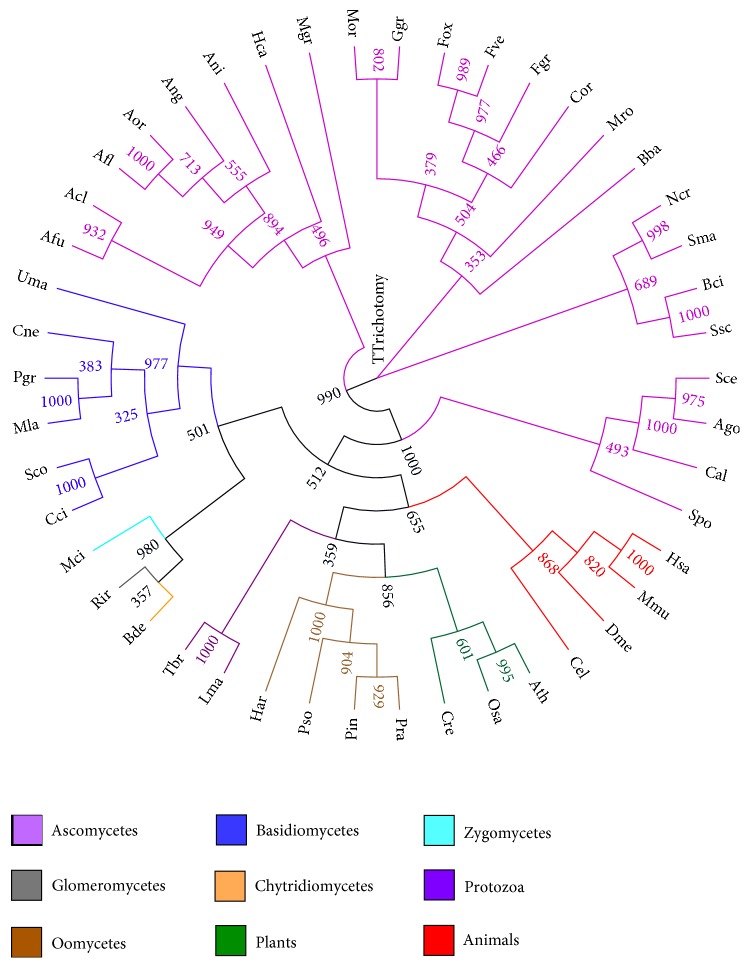
Phylogenetic tree of Rack1 proteins of eukaryotes. The predicted amino acid sequences of Rack1 proteins were aligned with ClustalX multiple sequence alignment program and the phylogenetic tree was visualized using FigTree program. Numbers indicate the 1000-bootstrap value of each branch. The accession number of each Rack1 protein is indicated in parentheses. Acl,* Aspergillus clavatus* (XP_001272169.1); Afl,* Aspergillus flavus* (EED47144.1); Afu,* Aspergillus fumigatus* (EDP50669); Ago,* Ashbya gossypii* (NP_985746.1); Ang,* Aspergillus niger* (XP_001389307.1); Ani,* Aspergillus nidulans* (XP_661767); Aor,* Aspergillus oryzae* (XP_001816748.1); Ath,* Arabidopsis thaliana* (NP_173248.1); Bba,* Beauveria bassiana* (XP_008600266.1); Bci,* Botrytis cinerea* (XP_001551314.1); Bde,* Batrachochytrium dendrobatidis* (XP_006679085.1); Cal,* Candida albicans* (P83774.2); Cci,* Coprinus cinereus* (XP_001830441.1); Cel,* Caenorhabditis elegans* (NP_501859.1); Cne,* Cryptococcus neoformans* (AAX94564.1); Cor,* Colletotrichum orbiculare* (ENH83707.1); Cre,* Chlamydomonas reinhardtii* (XP_001698065.1); Dme,* Drosophila melanogaster* (NP_477269.1); Fgr,* Fusarium graminearum* (XP_011318772.1); Fox,* Fusarium oxysporum* (KNB02916.1); Fve,* Fusarium verticillioides* (EWG39980.1); Ggr,* Gaeumannomyces graminis* (XP_009225866.1); Har,* Hyaloperonospora arabidopsidis* (HpaP813344); Hca,* Histoplasma capsulatum* (EGC41401.1); Hsa,* Homo sapiens* (NP_006089.1); Lma,* Leishmania major* (XP_001684560.1); Mci,* Mucor circinelloides* (OAD00838.1); Mgr,* Mycosphaerella graminicola* (XP_003852899.1); Mla,* Melampsora laricis-populina* (EGG07665.1); Mmu,* Mus musculus* (NP_032169.1); Mor,* Magnaporthe oryzae* (XP_003710816.1); Mro,* Metarhizium robertsii* (XP_007824428.1); Ncr,* Neurospora crassa* (CAA57460.1); Osa,* Oryza sativa* (NP_001043910.1); Pgr,* Puccinia graminis* (EFP89129.2); Pin,* Phytophthora infestans* (XP_002903189.1); Pra,* Phytophthora ramorum* (PSURA_41642); Pso,* Phytophthora sojae* (XP_009531042.1); Rir,* Rhizophagus irregularis* (ESA03866.1); Sce,* Saccharomyces cerevisiae* (NP_013834.1); Sco,* Schizophyllum commune* (XP_003029882.1); Sma,* Sordaria macrospora* (XP_003351440.1); Spo,* Schizosaccharomyces pombe* (AAA56865.2); Ssc,* Sclerotinia sclerotiorum* (EDN91497.1); Tbr,* Trypanosoma brucei* (XP_829201.1); Uma,* Ustilago maydis* (XP_011388829.1).

The Rack1 protein is found in many organisms, including the alga* Chlamydomonas reinhardtii*, yeasts, filamentous fungi, plants, nematodes, insects, and vertebrates, indicating that it is evolutionarily conserved [16, 58]. In fungi, Rack1 proteins are well conserved in most species of Chytridiomycota, Zygomycota, Glomeromycota, Ascomycota, and Basidiomycota (Figure 1). Rack1 proteins are also well conserved in fungi-like microbes, oomycetes, which cause serious plant diseases, such as potato late blight and sudden oak death [59, 60].

Rack1 proteins are functionally conserved. Multiple complementation assays show that Rack1 proteins are functionally interchangeable among different species, or even across kingdoms [26, 49, 52]. For example, the mammalian* RACK1* gene can rescue the defects of* rack1* mutants in* Schizosaccharomyces pombe *and* Aspergillus nidulans* [40, 52]. The* Neurospora crassa cpc-2* and the rat* RACK1* genes are able to complement the* cpc2* mutant in* S. pombe* [26].* RACK1* genes of* A. nidulans* and yeasts (*S. cerevisiae*,* S. pombe*, and* Candida albicans*) can largely rescue the hyphal growth defects of the* cpcB* deletion mutant in* Aspergillus fumigatus *[49]. Species-specific roles of Rack1 proteins have also been shown in different fungi. For example, the* A. nidulans CpcB* ortholog but not the* RACK1* genes from yeasts can completely rescue the conidiation defect of the* A. fumigatus cpcB* mutant [49]. Taken together, these studies show the well conserved and unique roles of Rack1 proteins in different fungal species.

## 3. Rack1 Proteins in Model Fungi

### 3.1. *Saccharomyces cerevisiae*

In* S. cerevisiae*, a model unicellular eukaryote, the Rack1 homolog, known as Asc1, is involved in multiple biological processes (Table 1). Asc1 is involved in the general amino acid control (GAAC) that provides the cell with sufficient amounts of protein precursors under conditions of amino acid limitation [61]. Amino acid starvation in yeast promotes translation of GCN4, which then causes expression of genes required for synthesis of all 20 amino acids. This response is called GAAC. In the absence of amino acid starvation, Asc1 represses Gcn4 [26], a transcription activator of the GAAC pathway [61]. In* S. pombe*, studies suggest that Cpc2 promotes the GAAC response under the amino acid starvation condition [41].

Asc1 functions as the G*β* subunit for Gpa2 and interacts directly with the G*α* subunit as a guanine nucleotide dissociation inhibitor that inhibits the guanine nucleotide exchange activity of G*α*. Asc1 negatively regulates glucose-mediated signaling via two pathways. In the cAMP/PKA pathway, Asc1 binds to the effector enzyme adenylyl cyclase (Cyr1) in addition to Gpa2 and represses the production of cAMP in response to glucose stimulation [27]. Secondly, Asc1 is involved in the Kss1 MAPK pathway by binding to Ste20. In the* asc1* mutant, basal phosphorylation of Kss1 is enhanced. Similarly, under pheromone stimulus, the* asc1* mutant shows enhanced phosphorylation of both Fus3 and Kss1 but shows reduced expression of gene* FLO11*, key components for pheromone response, mating, and filamentous growth [8, 28].

Asc1 is involved in cell wall integrity and stress responses [28–31]. The* asc1* mutant is hypersensitive to cell wall damaging agents, iron chelators, and nitrosative stress [29, 31]. Similarly, Cpc2 is involved in stress responses in* S. pombe* [42]. The cell wall integrity defect of the* asc1* mutant most likely results from PKC-independent mechanisms because the double-knockout mutant (*asc1 pkc1*) shows synergistic sensitivity to cell wall stresses [29]. Interestingly, the basal phosphorylation level of the MAPK Slt2 is higher in the* asc1* mutant [32], indicating that Asc1 regulates the* SLT2* MAPK pathway. In addition, Asc1 regulates replication stress-induced formation of P-bodies that consist of many enzymes involved in mRNA turnover [33, 62]. Asc1 also regulates cell size. Compared to the wild-type control, the cell size of the* asc1* mutant is larger in* S. cerevisiae* [28]. The enlarged cell size is also observed in the* cpc2* mutant in* S. pombe* [43].

Asc1 executes its many functions by interacting with different proteins and regulating the transcription and translation of different genes.* De novo* proteome analysis indicates that the* asc1* mutant shows 50% elevated* de novo* biosynthesis of soluble proteins, compared to the wild type. In contrast, expression of insoluble proteins is reduced by nearly a quarter in the* asc1* mutant [31]. The protein level of several transcription factors, including Rap1, Tec1, Phd1, and Flo8, is downregulated but the protein level of Ste12 is upregulated over 6-fold in the* asc1* mutant [31]. Interestingly, all three transcription factors, Flo8, Ste12, and Tec1, positively regulate the expression of gene* FLO11*. Flo8 and Tec1 are downregulated and Ste12 is upregulated in the* asc1* mutant. However, the expression of gene* FLO11* is still downregulated in the* asc1* mutant and the invasive growth is impaired [28]. Some proteins involved in cell wall biogenesis and overall cell morphology were found to be significantly reduced in the* asc1* mutant. Almost 50% of all proteins found to be regulated in* asc1* cells are involved in energy metabolism. This comprehensive group is composed of proteins taking part in glycolysis, mitochondrial biogenesis and respiration, oxidative stress, and fermentation. The findings of* de novo* proteome analysis are consistent with the phenotypes of the* asc1* mutant [31].

Asc1 is involved in translational regulation in* S. cerevisiae*. The Asc1 protein is a core component of the small (40S) ribosomal subunit [34–36]. Phosphoproteome and Western blotting studies show that the ribosomal protein Asc1 affects the phosphorylation of the eukaryotic translation initiation factors eIF2*α* and eIF4A and the ribosome-associated complex RAC [28]. Asc1 also participates in nascent peptide-dependent translation arrest at consecutive basic amino acid sequences, and the 40S subunit binding to Rack1 is crucial for translation arrest. Translation arrest of an mRNA by a nascent peptide leads to cleavage of the mRNA, as well as to cotranslational protein degradation [37]. Asc1 is required for efficient translation of short mRNAs with short open reading frames that usually show greater than average translational efficiency in diverse eukaryotes [38]. Asc1 is required to prevent frameshifting at ribosomes stalled at repeated CGA codons. In the absence of Asc1, ribosomes continue translation at CGA codons but undergo substantial frameshifting. Thus, the general translation fidelity of the cell depends upon Asc1-mediated quality control [39]. Taken together, these studies indicate that Asc1 constitutes a ribosomal interface for signal transduction and translational regulation [28].

### 3.2. *Neurospora crassa*

*N. crassa* is a model filamentous fungus [63]. In* N. crassa*, cross-pathway control (CPC) was first described by Carsiotis and collaborators [64, 65]. The cross-pathway control in* N. crassa* is the same as the GAAC in* S. cerevisiae*; that is, starvation for any one of several amino acids leads to globally increased synthesis of enzymes of many amino acid biosynthetic pathways in this fungus.* cpc-2* positively regulates the cross-pathway control under conditions of amino acid limitation [46]. Therefore, there is no activation of target amino acid biosynthetic genes in the* cpc-2* mutant under starvation conditions (Table 1). Under nonstarved conditions, mutation of the* cpc-2* gene decreases fungal growth. Formation of protoperithecia during sexual reproduction is impaired in the* cpc-2* mutant [47]. Genetic epistasis between* cpc-2* and components of the G protein pathway has been studied [48]. One Ga subunit (*gna-3*) is epistatic to* cpc-2* during submerged-culture conidiation, while two other G*α* subunits (*gna-1*,* gna-2*) are independent of* cpc-2*, and the G*β* (*gnb-1*) and G*γ* (*gng-1*) subunits operate downstream of* cpc-2* during submerged-culture conidiation. In yeast two-hybrid assays,* gna-3* interacts with* cpc-2*. The epistatic studies together with the protein-protein interaction studies indicate that* cpc-2* does work in the heterotrimeric G protein complex.

## 4. Rack1 Proteins in Pathogenic Fungi

### 4.1. *Candida albicans*

*C. albicans* is an opportunistic pathogen and causes life-threatening systemic infections in immunocompromised individuals [66]. Its virulence factors include host recognition biomolecules (adhesins), morphogenetic transitions (the reversible transition between unicellular yeast cell and filamentous growth forms), biofilms, secreted aspartyl proteases, and phospholipases [44, 66]. From transcriptomics and proteomics data, Liu et al. show that the expression of* ASC1* in* C. albicans* is iron-, temperature-, and Gcn4-dependent and is downregulated by amino acid starvation, caspofungin, and farnesol (Table 1) [45]. The* asc1* null mutant displays shorter hyphae on several liquid and solid hypha-inducing media, compared to the wild-type strain [45]. Adhesive growth and invasive growth are impaired in the* asc1* null mutant [44]. The* asc1* null mutant has attenuated virulence. In a mouse model of systematic infection, the* asc1* null mutant is dramatically less virulent than the wild-type strain shown by the fact that 50% of the mice infected with the* asc1* null mutant survived, while the wild-type strain killed all the infected mice. In another assay using a mouse model of disseminated infection, nearly all of the mice inoculated with the wild-type strain died within 16 days. However, mice infected with the* asc1* null mutant were completely asymptomatic [44]. Finally, Fan et al. show that transcription of adhesion-related genes* ALS3*,* ECE1*, and* HWP1* [67] is reduced in the* asc1* null mutant, indicating that Asc1 regulates virulence of* C. albicans* through these important genes [44].

### 4.2. *Aspergillus nidulans* and* Aspergillus fumigatus*

*A. nidulans*, a model filamentous fungus, is also reported as an opportunistic pathogen [68, 69].* A. fumigatus*, the major causative agent of life-threatening invasive aspergillosis in immunocompromised individuals, is a ubiquitous saprophytic fungus [70]. Thermotolerance, specialized cell wall, melanization, resistance to oxidative stress, and mycotoxins (gliotoxin) are important for virulence of* A. fumigatus*. In addition, the cAMP/PKA pathway regulates the synthesis of several virulence factors [71]. In both* A. nidulans *and* A. fumigatus*, Rack1 proteins, CpcB, play important roles in fungal growth, conidiation, conidial germination, and mycotoxin production (Table 1) [49, 50]. In* A. nidulans*, CpcB represses the cross-pathway control in the presence of amino acids and regulates sexual development [50, 52]. Compared to the wild-type strain, the* cpcB* mutant of* A. nidulans *produces much fewer cleistothecia, and the cleistothecia contain no ascospores, indicating that CpcB is required for proper formation of fruiting bodies and ascosporogenesis [50, 52]. In* A. fumigatus*, CpcB is localized in the cytoplasm. To determine the genetic relationship between CpcB and GpaB (Ga), the* gpaB* mutant and the double-deletion mutant,* cpcB gpaB*, were generated. The double mutant* cpcB gpaB* causes a similar phenotype to the* gpaB* mutant with abnormal multiple septa conidiophores, indicating the overlapping functions of CpcB and GpaB [49]. Cell wall integrity of the* cpcB* mutant is impaired, revealed by transmission electron microscopy examinations [49]. Virulence of the* cpcB* mutant is attenuated in an immunosuppressed mouse model. The study also demonstrates that the G–H residues in WD repeats 1, 2, and 3 and the W–D residue in WD repeat 2 of CpcB are required for normal hyphal growth and conidiation and are also necessary for maintaining normal antifungal drug susceptibility. The* cpcB* mutant displays enhanced drug resistance, which might be due to reduced intracellular drug accumulation and altered ergosterol component. In addition, the first G–H residue of CpcB plays a critical role in the virulence of* A. fumigatus* [51]. The involvement of CpcB in pathogenesis of* A. fumigatus* might be through its connection with the G protein complex and/or its role in maintaining proper cell wall structure.

### 4.3. *Cryptococcus neoformans*

*C. neoformans* is an encapsulated yeast-like basidiomycetous fungus.* C. neoformans* has a worldwide distribution [72]. It is found in soil, bird excrement, and trees. It is an opportunistic pathogen and causes meningoencephalitis in immunocompromised individuals [73]. Fungal capsulation and melanization, among others, are important for its virulence. The major virulence factors of the pathogen are regulated by the cAMP/PKA pathway [53]. In* C. neoformans*, the Rack1 protein Gib2 is required for full virulence (Table 1) [53–55]. Gib2 functions as a scaffolding adaptor protein and interacts with G*α* (Gpa1) and G*γ* (Gpg1 and Gpg2) proteins. Gib2 also interacts with the protein kinase C1 Smg1, a phosphodiesterase Pde2, and the small G protein Ras1, which are all involved in the cAMP/PKA pathway. Gib2 positively regulates the cAMP/PKA pathway, for overexpression of Gib2 rescues the defects of the* gpa1* mutant in both melanization and capsule formation and restores the cAMP level by relieving the inhibitory effect of Ras1 on the adenylyl cyclase Cac1 in the* gpa1* mutant [54, 55]. In a murine model of cryptococcosis, disruption of the* GIB2* gene results in severe attenuation of virulence [54]. In summary, Gib2 is involved in virulence by acting as a scaffolding adaptor protein of the cAMP/PKA pathway in* C. neoformans*.

### 4.4. *Ustilago maydis*

*U. maydis*, a dimorphic basidiomycete, causes corn smut and serves as a model for obligate biotrophic fungal pathogens [74]. Infection of maize by* U. maydis* requires that haploid yeast cells of compatible mating types fuse and establish dikaryotic filamentous hyphae. The morphological transition from budding to filamentous hyphae is important for fungal virulence and is regulated by the conserved cAMP/PKA and MAPK pathways. Loss of Rack1 protein, Rak1, causes slow growth, hypersensitivity to cell wall stress, attenuated cell fusion, and attenuated virulence in* U. maydis* (Table 1) [57]. In infection assays, more than 80% of plants infected with the wild-type strain showed tumor formation but no tumors could be observed in* rak1*-infected maize plants. Filament formation and appressorium formation are impaired in the mutant. The attenuated cell fusion likely results from the reduced expression of* rop1*, a transcriptional activator of the pheromone response factor (prf1) that regulates pheromone (mfa) and pheromone-receptor genes. Rak1 is a ribosome-associated protein and interacts with a large number of proteins including 32 ribosomal proteins shown in protein pull-down assays. These proteins are involved in metabolism, energy production, cell division, DNA replication, rRNA processing, and UDP-galactose translocation, which are possibly involved in fungal pathogenesis [75]. The constitutive expression of* rop1* or constitutive activation of the pheromone-responsive MAPK pathway could rescue the defect of the* rak1* mutant in conjugation tube formation. Prf1 is regulated at the posttranscriptional level by the cAMP/PKA pathway and the MAPK pathway, indicating that Rak1 possibly functions in the cAMP/PKA and MAPK pathways [74]. However, no interaction between Rak1 and any G*α* subunits has been established, and additional studies are needed to determine how Rak1 participates in the cAMP/PKA and MAPK pathways that are required for pathogenicity of* U. maydis*.

## 5. Concluding Remarks and Perspectives

Rack1 proteins play important roles in fungal pathogenesis. However, compared to the intensive studies on Rack1 proteins in human, plants, and yeasts [12, 16, 39], limited studies have been conducted in a few pathogenic fungi. In these fungi, Rack1 proteins affect various virulence factors through conserved signal transduction pathways (Table 1) [76, 77]. In other fungal pathogens, such as* Penicillium marneffei* and* Histoplasma capsulatum*, Rack1 proteins are found to be associated with stress responses or dimorphic transition [78, 79]. In plant pathogens* Fusarium verticillioides* and* F. graminearum*, WD40 proteins are required for virulence and other important functions, though the Rack1 proteins have not been characterized [80]. In addition, protein-protein interaction network analyses indicate that WD40 proteins are highly connected in the malaria parasite, with 1,928 potential interactions, supporting the role of WD40-containing proteins, including Rack1 proteins, as hubs in cellular networks [81]. Given the conserved and versatile roles of Rack1 proteins in eukaryotes [34], it is important to explore the unique and dynamic roles of Rack1 proteins in different pathogenic fungi.

## Figures and Tables

**Table 1:** Rack1 proteins in model and pathogenic fungi.

Species	Rack1	Functions	References
Ascomycetes			
*Saccharomyces cerevisiae*	Asc1	General amino acid control, pheromone response, mating and filamentous growth, pseudohyphal development, translation regulation and fidelity, cell size, cell wall integrity, stress responses, and cAMP/PKA and MAPK pathways	[8, 26–39]
*Schizosaccharomyces pombe*	Cpc2	Stress responses, cell wall integrity, cell size, mitotic commitment, positive role in the general amino acid control response, cell cycle progression, and meiotic development	[40–43]
*Candida albicans* ^*∗*^	Asc1	Growth, filamentation, adhesive growth, invasion, and virulence	[44, 45]
*Neurospora crassa*	*cpc-2*	Cross-pathway control, sexual reproduction, and the heterotrimeric G protein complex	[46–48]
*Aspergillus fumigatus* ^*∗*^	CpcB	Growth, conidiation, conidial germination, cell wall integrity, mycotoxin (gliotoxin) production, virulence, and antifungal drug resistance	[49–51]
*Aspergillus nidulans*	CpcB	Growth, conidiation, conidial germination, sexual development, mycotoxin (sterigmatocystin) production, and cross-pathway control	[50, 52]
Basidiomycetes			
*Cryptococcus neoformans* ^*∗*^	Gib2	Growth, virulence, a scaffolding adaptor protein, and a G*β*-like subunit of the cAMP/PKA pathway	[53–56]
*Ustilago maydis* ^§^	Rak1	Growth, cell wall integrity, cell fusion, filament formation, appressorium formation, virulence, and a ribosomal protein	[53, 57]

^*∗*^Human pathogens.

^§^Plant pathogens.
